# School climate and left-behind children’s achievement motivation: The mediating role of learning adaptability and the moderating role of teacher support

**DOI:** 10.3389/fpsyg.2023.1040214

**Published:** 2023-01-23

**Authors:** Keyun Zhao, Ning Chen, Guanling Liu, Zhijun Lun, Xinghua Wang

**Affiliations:** ^1^School of Communication, Qufu Normal University, Rizhao, China; ^2^Normal College, Qingdao University, Qingdao, China

**Keywords:** left-behind children, school climate, teacher support, learning adaptability, achievement motivation

## Abstract

School climate has been reported to have an important impact on children’s achievement motivation, but the mechanism for the impact of school climate on left-behind children has not been fully explored. The purpose of this study is to investigate the roles of left-behind children’s learning adaptability and teacher support in mediating and moderating the relationship between school climate and achievement motivation. In this study, 1,417 left-behind children were surveyed. The results showed that: (1) after controlling for gender and age, the school climate still had a positive effect on the achievement motivation of left-behind children (*c′* = 0.177, *p* < 0.001). (2) School climate perceived by left-behind children directly predicted their achievement motivation, and indirectly through their learning adaptability (*a*1 = 0.338, *p* < 0.001; *b* = 0.341, *p* < 0.001). In other words, left-behind children’s learning adaptability may play an intermediary role between school climate and achievement motivation. (3) The indirect effect of school climate on achievement motivation through learning adaptability was moderated by teacher support (*a*2 = 0.153, *p* < 0.001), and this indirect effect was more significant for left-behind children who perceived high teacher support. The research reveals the importance of school climate and teacher support to the growth and development of left-behind children, thus holding theoretical significance for improving the achievement motivation of left-behind children.

## 1. Introduction

As an important part of the social growth of individuals, achievement motivation was originally called “Need to achievement,” which was proposed by psychologist Murray (1938) in his book “Investigation of Personality.” Later, McClelland et al. (1953) borrowed Murray’s concept. He formally proposed the Achievement Motive, which he defined as: “a need or drive for success learned by an individual in competition with his good principles (McClelland et al., 1953). “He also points out that an individual’s childhood experience can have an impact on his motivation to achieve. As a special group of left-behind children in the process of social development, achievement motivation is the power source for the stability and development of their internal psychological and personality characteristics (Fini and Yousefzadeh, 2011), which is the internal power to ensure the healthy growth of left-behind children. For left-behind children, their parents went to other places to work, causing the alienation of the parent–child relationship and the lack of family functions. In addition to the family environments, school as the main place for children to study and live may replace some functions and responsibilities of the family. It plays a key role in making up for the lack of family support functions for left-behind children (Wang, 2018).

Although studies (Pescaru, 2015; Tan et al., 2020; Zhou et al., 2022) have identified the influence of many family factors on left-behind children, very few have investigated the role of school factors on the growth and development of left-behind children. As the main place for children’s study and life, it is necessary to conduct an in-depth exploration of schools. School climate is a key factor in the school, and some researchers have explored the role of the school climate on children’s achievement and development (Aişi Lăzărescu, 2012; Maxwell et al., 2017). In addition, the school climate has a more significant influence on left-behind children (Wang, 2018). Prior studies suggest that the school climate has a significant impact on students’ achievement motivation (Anderson, 1982; Cohen et al., 2009). A good school climate positively promotes the social–emotional, and achievement development of adolescents (De Pedro et al., 2016). However, some researchers have pointed out that it is not enough to discuss the direct relationship between variables, and that potential mediating variables need to be introduced to answer the question of how campus climate works (Johnson and Stevens, 2006; Loukas and Murphy, 2007). Learning adaptability refers to the ability of learners to adapt to the school environment and learning activities under the school climate. Adequate learning adaptability can have a significant impact on the development of personal achievement. Previous studies have shown that learning adaptability has a significant mediating effect on the relationship between external environmental factors and student development (Zhao et al., 2020). This study intends to further explore whether the mediating effect of learning adaptability is significant in the relationship between school climate and achievement motivation. Therefore, this study introduced the variable of learning adaptability, used it as a mediation variable between the school climate and achievement motivation, and examined its mediation effect on the relationship between them.

As the behavioral subject of school development (Furman and Buhrmester, 1992), teachers not only play an important role in school education and teaching but also are one of the main factors affecting the development of students’ cognitive and noncognitive abilities. The supporting role of teachers can buffer the effects of family adverse factors caused by migrant parents on students’ academic performance (Luthar et al., 2000), which is the key factor affecting the achievement and development of left-behind children in rural areas. At the same time, high-quality teacher support can enhance students’ understanding of ability and learning autonomy, to enhance their achievement motivation and learning participation (Lee and Simpkins, 2021). To explore whether different levels of teacher support have different effects on the relationship between school climate and learning adaptability, this study introduced teacher support as a variable to explore its mechanism.

The studies above have shown that school factors have an important influence on the achievement motivation of left-behind children. Therefore, this study is necessary to further explore the potential mechanisms of school climate on achievement motivation development of left-behind children. We hope to find a direct or indirect relationship between the school climate and the performance motivation of left-behind children, and to determine the intermediary effect of learning adaptability and the adjustment effect of teacher support. To address this issue, this study used the PROCESS plugin in the SPSS software to fill the gaps in the literature by testing mediation variables (i.e., learning adaptability) and adjustment variables (i.e., teacher support for school climate and learning adaptability).

### 1.1. The relationship between school climate and achievement motivation

Schools are one of the places where students develop close relationships with others beyond their family ties (Bronfenbrenner and Evans, 2000; Eccles and Roeser, 2011). School climate is a relatively stable and lasting environmental feature that members of the school can perceive in terms of organization, teaching, and interpersonal relationships (Roeser et al., 2000; Cohen et al., 2009). Self-determination theory is a kind of motivational process theory about human self-determined behavior. It was developed by American psychologists Deci Edward L. and Ryan Richard M. in the 1980s. According to self-determination theory, the external environment can enhance the internal motivation of individuals, promote the internalization of external motivation and ensure the healthy growth of individuals by supporting the satisfaction of the three basic psychological needs of autonomy, competence, and relatedness (Deci and Ryan, 2000a,b). It can be seen that the theory emphasizes the role of the situation and its role in satisfying individual needs and points out that the degree to which basic psychological needs are satisfied affects the effective execution of various tasks. According to this view, school, as an important external environment for children’s growth, can create the best conditions to meet students’ basic needs (Kulakow, 2020), thereby enhancing children’s motivation to pursue success. It can be seen that a good school atmosphere helps to improve children’s achievement motivation. The literature suggests that a good school environment can affect adolescent motivation in many ways (Cohen et al., 2009). For example, school culture (Wang M. T. et al., 2022), campus relationships (Anderson, 1982), and classroom environments (Alonso-Tapia and Ruiz-Díaz, 2022) are all related to high achievement motivation (Doğan and Sezer, 2010). Adolescents perceive the school environment as contributing to personal achievement motivation when it provides clear expectations, consistency and predictability of responses, emotional support, opportunities to learn and master meaningful material, and adequate or appropriate support for students’ individual goals and interests, Students’ academic self-concept and subjective task attention have been improved (Wang and Eccles, 2013). Based on the research above, it can be concluded that an effective and active school climate can facilitate the implementation of school learning activities and the realization of educational goals so that students can achieve success. Therefore, the following hypothesis is proposed:

*H1*: School climate has a positive predictive effect on the achievement motivation of left-behind children.

### 1.2. Mediating role of learning adaptability

Learning adaptability refers to the ability of individuals to make appropriate adjustments to their behaviors, cognition, or emotion when facing uncertainties in the learning process (Martin et al., 2013). Previous studies (Liu et al., 2014; Stockinger et al., 2021; Şahin and Gülşen, 2022) have shown that learning adaptability plays an important role in students’ development. Learning adaptability significantly predicts students’ self-efficacy, academic achievement, and self-regulated learning behaviors (Martin, 2017; Burns et al., 2018). Students with stronger learning adaptability can better deal with uncertain factors in their learning environments by generating positive achievement motivation and actively responding to the challenges of the environments (Collie et al., 2017). The study above discussed the importance and practical value of learning adaptability. This study chose learning adaptability as the intermediary variable. The main reason is that learning adaptability is the internal driving force for the development of adolescent achievement motivation in the school climate (Zhao et al., 2020). For left-behind children, due to the lack of family support, their schools assume the main educational responsibilities. Proper learning adaptability is helpful for left-behind children to rapidly integrate into a new community, facilitating the development of positive interpersonal relationships and the formation of learning motivation (Mu and Zhao, 2015).

Based on self-determination theory, meeting innate psychological needs is believed to be essential for students’ development and well-being (Deci and Ryan, 2000a). Self-determined motivation was positively predicted by the satisfaction of psychological needs and negatively predicted by the thwarting of psychological needs (Trigueros et al., 2019). A good school climate is conducive to meeting the basic needs of individuals for competence, autonomy, and relatedness (Kulakow, 2020), and meeting these needs has an important impact on individual adaptability. In education, research has proved that the psychological needs of students are met with various aspects of adaptation. Students’ basic psychological needs are closely related to students’ participation, academic self-efficacy, career adaptability and other adaptive outcomes (Şahin and Gülşen, 2022). Studies have shown that perceived psychological needs predict higher academic adjustment scores, For example, deep learning method, high level of intrinsic motivation (Doménech-Betoret and Gómez-Artiga, 2014), high level of academic adaptation, adaptive learning strategy, active classroom participation (Ratelle and Duchesne, 2014; Sulea et al., 2015). Unmet basic psychological needs are the main reasons for the decrease in students’ learning interest and learning engagement, their aversion to school life, and their learning burnout (Luo et al., 2014; Maralani et al., 2016), which are all manifestations of learning maladjustment in children. Therefore, based on self-determination theory, when the school provides a supportive environment to meet the individual’s needs for autonomy, competence, and relatedness, these variables further promote the emergence of students’ learning adaptive behaviors. Some studies can prove that students’ perception of school climate is significantly related to their learning adaptability (Kuperminc et al., 2001). Several studies have found that student behaviors and learning performance can be predicted by different classroom climates (Alonso-Tapia and Ruiz-Díaz, 2022), which can improve student participation in learning and allow students to adapt better (Eccles, 2004; Roorda et al., 2011). Moreover, positive interpersonal relationships in the school climate are also the main factors that promote learning adaptability (Lee et al., 2019). According to the self-determination theory (Deci and Ryan, 2000b), when left-behind children perceive the care from their teachers and classmates, their basic psychological needs are likely to be met, and they can develop the agency and capacities to cope with challenges and solve problems (Hawkins et al., 2001; Amemiya and Wang, 2018).

To sum up, a positive school climate will promote the development of students’ learning adaptability, and subsequently affect the development of their achievement motivation. Therefore, this study proposes the following hypotheses:

*H2*: School climate has a positive predictive effect on the learning adaptability of left-behind children.

*H3*: The learning adaptability of left-behind children can significantly predict their achievement motivation.

### 1.3. The moderating role of teacher support

Teacher support refers to students’ perception of teachers’ care and helps as well as their trust in teachers (Trickett and Moos, 1973; Ryan and Patrick, 2001). To reveal the effect mechanism of school climate on the motivation of left-behind children, this study focuses on the “condition” of variable relationships based on constructing an intermediary action model of learning adaptability.

By adding a moderating variable (i.e., teacher support), the study built a moderated mediation model to investigate how the interplay between school climate and teacher support affects the development of left-behind children. Research (Quin et al., 2018; Pap et al., 2021) shows that teachers can increase students’ learning engagement and motivation through supportive behaviors and improve their academic performance. In addition, the mentoring role of teachers can contribute to the development of children’s academic achievement (Trigueros et al., 2020). In the absence of parental support, teacher support helps left-behind children maintain enthusiasm for learning (Hardre and Reeve, 2003). Teachers are considered important persons in the life of left-behind children, whose concern and encouragement can enable left-behind children to fully tap their potential (Liu et al., 2021). Meanwhile, the more teachers support left-behind children perceive, the easier it is for them to adapt to school life and the higher their learning adaptability will be (Zhang et al., 2021). Following this line of argument, it can be seen that teacher support has an important impact on the physical and mental development of left-behind children and the formation of achievement motivation.

In addition, social support theory suggests that individuals’ perceived supportive behaviors from their social networks are universally gainful and contribute to individuals’ psychological health and development (Ma et al., 2021). If individuals perceive that their organization promotes a healthy learning culture through feedback and guidance, their motivation to learn and share new knowledge will be further enhanced (Banerjee et al., 2017). As such, it can be argued that if left-behind children believe their teachers forge a genuine learning culture, they will continue to strengthen their sense of belonging to their schools, which ultimately contributes to their motivation to pursue academic success. In addition, teachers are important supporters of students in the school environment, and their supportive behaviors influence how students’ basic psychological needs are met. Prior research (Wang Q. et al., 2022) has shown that students show higher levels of motivation when their psychological needs are met by the environment in which they live. A favorable school climate is conducive to the satisfaction of students’ basic psychological needs. The more support the left-behind children get from their teachers, the more likely it is for them to develop trust and a sense of belonging to their teachers and schools, and the higher their willingness to achieve academic success. Therefore, this study proposes the following two hypotheses:

*H4*: Teacher support plays a moderating role in the relationship between school climate and left-behind children’s achievement motivation. When teacher support is high, school climate has a greater influence on their achievement motivation.

*H5*: Teacher support plays a moderating role in the relationship between school climate and left-behind children’s learning adaptability. When teacher support is high, school climate has a greater impact on their learning adjustment.

**Figure 1:**
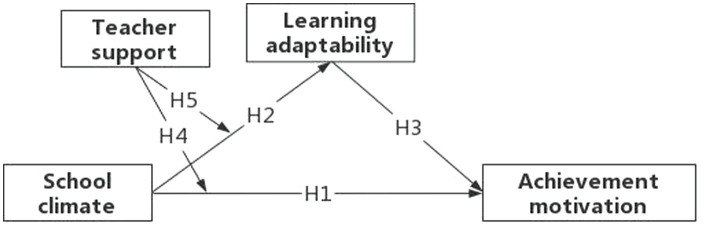
Conceptual model diagram.

Based on the above analysis, the study developed a moderated mediation model, which is illustrated in Figure 1. The main purposes are two-fold: (1) to investigate the possible mediating role of learning adaptability between school climate and achievement motivation of left-behind children; (2) to verify whether the teacher support adjusts the mediation model. This model could deepen our understanding of the relationship between school climate and achievement motivation. It not only reveals how school climate is related to the achievement motivation of left-behind children but also helps identify how teachers’ support could regulate the relationship between the two factors.

## 2. Methodology

### 2.1. Participants and research setting

This study investigated 1,417 left-behind children in rural areas of Gansu Province, China. As this province’s economy is relatively backward, there are a large number of local farmers and workers going out to work. Several primary schools and middle schools were approached through the local educational bureau. Their permission was obtained. Four classes from each grade in the schools were randomly selected to fill out the survey. According to local government standards, left-behind children in rural areas refer to those under the age of 18 who have one or both parents working outside for at least 6 months (Yang and Liu, 2020; Wang and Liu, 2022). Children were selected according to the following inclusion criteria: (1) children are younger than 18 years old; (2) one or both of the parents of children have been away for at least 6 months. The demographic information is shown in Table 1. The study included 664 male and 753 female participants aged between 9 and 17 (*M* = 12.05, SD = 1.81). According to the situation of parents’ mobility, left-behind children can be divided into three types: left behind with father (*N* = 83, 5.9%), left behind with mother (*N* = 575, 40.6%), and left behind with grandparents (*N* = 759, 53.6%). In terms of education levels, the students are divided into three categories: primary education (no schooling, primary school), secondary education (junior high school, senior high school, or technical secondary school), and higher education (university and above). 54.7% of respondents’ fathers had received primary education, 41.6% had received secondary education and 3.7% had received higher education, while 62.9% of respondents’ mothers had received primary education, 33.6% had received secondary education and 3.5% had received higher education.

**Table 1:** Means, standard deviations, and correlations among variables.

	*M* (SD)	1	2	3	4	5
1. Gender	1.53 (0.50)	-	-	-	-	-
2. Age	12.05 (1.81)	0.024	-	-	-	-
3. School climate	4.01 (0.63)	−0.009	−0.099***	-	-	-
4. Achievement motivation	3.92 (0.53)	0.016	−0.094***	0.475***	-	-
5. Learning adaptability	4.08 (0.66)	0.036	−0.095***	0.606***	0.561***	-
6. Teacher support	4.44 (0.62)	−0.001	−0.056*	0.765***	0.459***	0.608***

Gender is a dummy variable, male = 1, female = 2; ***p < 0.001, *p < 0.05.

The study was approved by the Institutional Review Committee. Before conducting the survey, the researchers obtained the informed consent of the participants’ caregivers and guardians. The data collection process was completed during school hours in an online classroom environment. The head teachers supervised the whole process by explaining the purpose and details of the study and answering any questions participants had as they filled out the questionnaire. Participants were told there was no right or wrong answer and were allowed not to discuss the topic with each other, and each completed four questionnaires. Participants took about 25 min to complete the test.

### 2.2. Instrumentation

#### 2.2.1. School climate

The school climate scale (Li et al., 2016) was used to measure students’ perceptions of school climate. After factor analysis of the original scale, items with factor loadings lower than 0.40 were deleted from the scale (Pituch and Stevens, 2015). The final scale included four dimensions: teacher-student relationship (e.g., “Teachers often care about our daily lives”); Student–student support (e.g., “I get on well with my classmates”); Teaching atmosphere (e.g., “The school often holds various competitions”); Learning atmosphere (e.g., “Everyone in the class likes to study”). Each item on the scale was rated on a 5-point Likert scale (1 = “completely disagree,” 5 = “completely agree”). The scores of the scale were calculated after the item scores were averaged. The higher the total score was, the more positive the school climate would be. This scale has been widely used in the context of China (Zhang et al., 2020). In this study, the Cronbach’s alpha coefficient of this scale was 0.913, which was acceptable.

#### 2.2.2. Learning adaptability

The study mainly used the learning adaptability scale developed by Feng et al. (2006) to evaluate the learning adaptability of the subjects. There were 9 questions in total, including 4 questions from the dimension of learning attitude (such as “I never ignore difficulties in learning”) and 5 questions from the dimension of learning ability (such as “I can solve new problems with what I have learned”). Each item was rated on a 5-point Likert scale (1 = “completely disagree,” 5 = “completely agree”). Previous studies have proved that this scale has good reliability and validity and can effectively measure students’ learning adaptability (Liu et al., 2014). In this study, Cronbach’s alpha coefficient was 0.899, indicating that the revised scale had adequate reliability.

#### 2.2.3. Teacher support

The perceived teacher support scale developed by Wong et al. (2018) was used. The scale included two dimensions: instrumental support (e.g., “When I meet difficulties in learning, the teacher can help me in time”) and evaluative support (e.g., “The teacher can find our bad habits and help us correct them”). Each item was rated on a 5-point Likert scale (1 = “completely disagree,” 5 = “completely agree”). The scale score was calculated after the item scores were averaged, with the higher score indicating that children perceived higher support from teachers. Previous studies have confirmed that the scale has high reliability and validity and can reflect teenagers’ perception of teacher support (Sadoughi and Hejazi, 2021). In this study, Cronbach’s alpha coefficient of the teacher support scale was 0.914, implying a satisfactory internal consistency and acceptable validity.

#### 2.2.4. Achievement motivation

The achievement motivation scale (Yu and Yang, 1987) was developed to evaluate achievement motivation. This scale has been widely applied in the measurement of Chinese children and adolescents (Zhang, 1998). The scale includes two dimensions, social-oriented achievement motivation and personal-oriented achievement motivation, and contains a total of 37 questions. The 5-point Likert scale was used to score each item on the scale (1 = “completely inconsistent,” 5 = “completely consistent”). The higher the average score of each dimension, the higher the corresponding “social-oriented” and “individual-oriented” achievement motivation would be. In this study, the scale showed high reliability, with Cronbach’s alpha value of 0.921.

### 2.3. Data analysis

The effective data collected were analyzed by SPSS 24.0 software. Firstly, the Harman univariate test was used to test the existence of common method bias. Descriptive statistics and correlation analysis were conducted for the six variables to determine the relationship between the variables. In addition, collinearity diagnosis was performed to confirm the existence of multiple collinearities among variables. Secondly, model 8 in PROCESS version 3.5 of SPSS 24.0 macro program was used to test the mediating effect of learning adaptability and the moderating effect of teacher support after controlling for gender and age. Results show that the control two variables of gender and age, the school atmosphere and achievement motivation between the direct and indirect influence of left-behind children learning adaptability in school plays a role of intermediary between atmosphere and achievement motivation, learning adaptability and the intermediary variable in the intermediary role between the two by regulating variable regulation of teachers’ support. Moreover, teacher support plays a moderating role in the relationship between school climate and achievement motivation. Finally, in order to further understand the regulation of moderating variables on the model, the point selection method and Johnson-Neyman (Dearing and Hamilton, 2006) method were used to test the simple slope, so as to have an in-depth understanding of the moderating effect of different levels of moderating variables on the model.

## 3. Results

### 3.1. Common method bias and multicollinearity diagnosis

On the one hand, the common method deviation is controlled by designing a partial reverse problem and adopting an anonymous measurement method. On the other hand, the common method bias is controlled by cross-validation of partial problems with different forms of the same problem. After data collection, the common method deviation was controlled from the aspect of statistical control by analyzing the non-rotating exploratory factor analysis results. The results were as follows: KMO and Bartlett Test of Sphericity results was 0.965 (*p* < 0.001), indicating that it was suitable for the factor analysis. Then 13 factors with characteristic roots greater than 1 were extracted by principal component analysis, and the cumulative variance explanation rate of the first factor was 26.51% (less than 50%; Podsakoff and Organ, 1986), indicating that the common method deviation was not serious and the data analysis results were reliable.

In addition, to confirm the presence of multicollinearity between variables, collinearity diagnosis was performed. The results show that the inflation factor (VIF) values of all variances are far less than the critical value of 10, and the tolerance is greater than 0.1. Therefore, there is no multicollinearity problem in the model (Chennamaneni et al., 2016).

### 3.2. Preliminary analysis

Table 1 presents descriptive statistics and correlation analysis results of demographic variables and each observation variable. Therefore, in the survey results with a sample size of 1,417, the variables of school climate, achievement motivation, learning adaptability, and teacher support were all positively and significantly correlated with one another, with the correlation coefficients ranging from 0.475 to 0.765. The age of left-behind children was negatively correlated with school climate, achievement motivation, learning adaptability, and teacher support. This result shows that the perceived school climate and teacher support could decrease with the increase in children’s age. This is mainly because left-behind children become mentally mature and their independence increases as they grow old, and their dependence on the school environment and school teachers will decrease. In addition, as children advance in grade, they face more life and physical problems. Multiple reasons, such as heavy homework burden, fierce competition among classmates, strict requirements of teachers, and extra attention to academic performance (Chaudhuri et al., 2022; Granziera et al., 2022; Kong and Lin, 2022), will lead to low attention of left-behind children to teachers. At the same time, as left-behind children get older, their learning adaptability and achievement motivation may also decrease. The reason could be that left-behind children in lower grades have easier academic tasks and less learning pressure, which enables them to maintain a positive attitude in life and study, thus promoting the development of learning adaptability and achievement motivation. Due to the significant correlation between age and school climate, learning adaptability, and achievement motivation, age was controlled in the subsequent moderating effect analysis. Although there is no significant correlation between gender and the variables of school climate, learning adaptability, and achievement motivation in the current correlation analysis, the variable of gender can have a certain impact on achievement motivation in previous studies (Kumari and Sangwan, 2021). Therefore, the gender of left-behind children was also controlled in the follow-up examination.

### 3.3. Moderated mediation model testing

**Figure 2:**
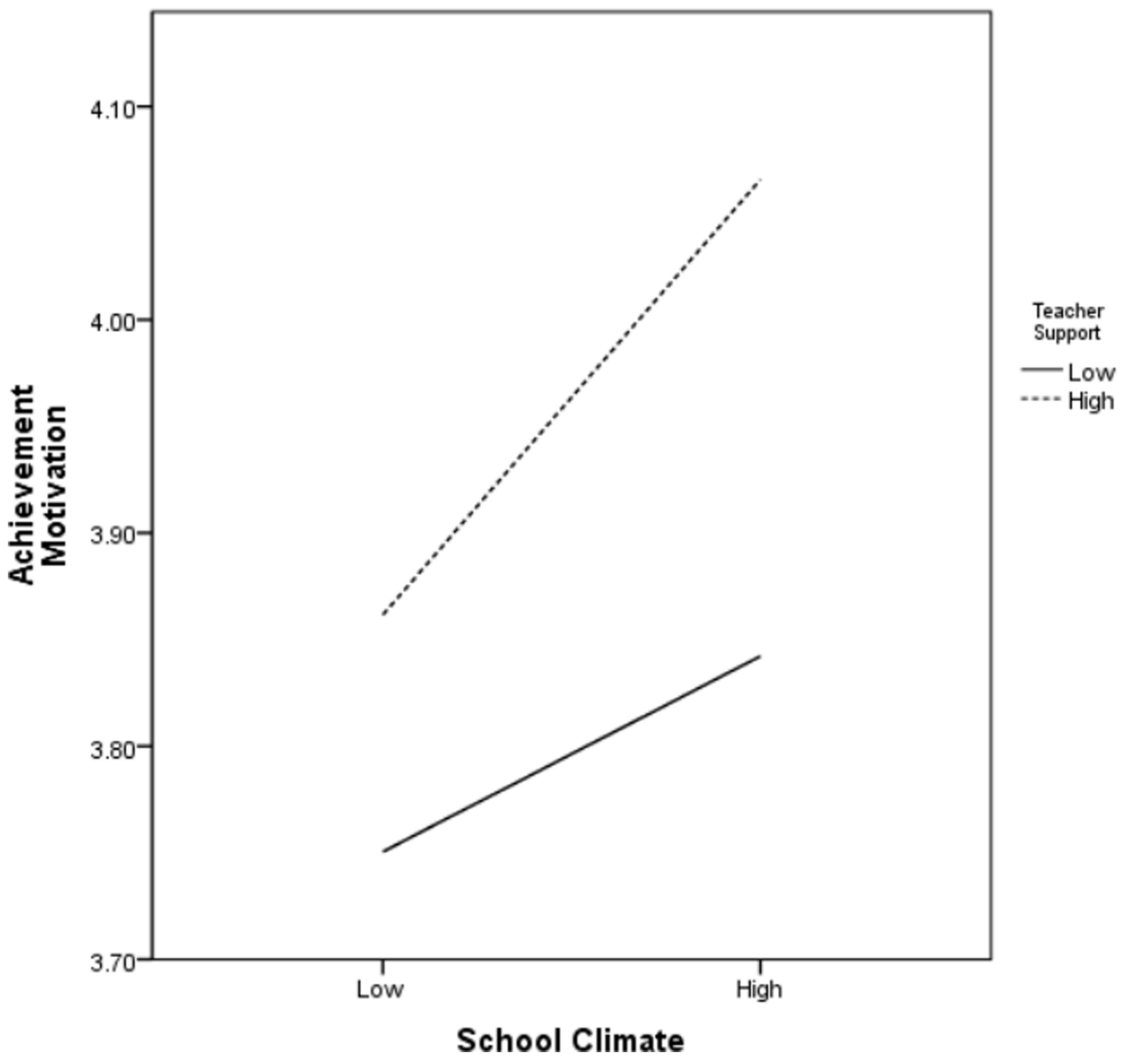
Moderating effects of teacher support on school climate and achievement motivation.

**Figure 3:**
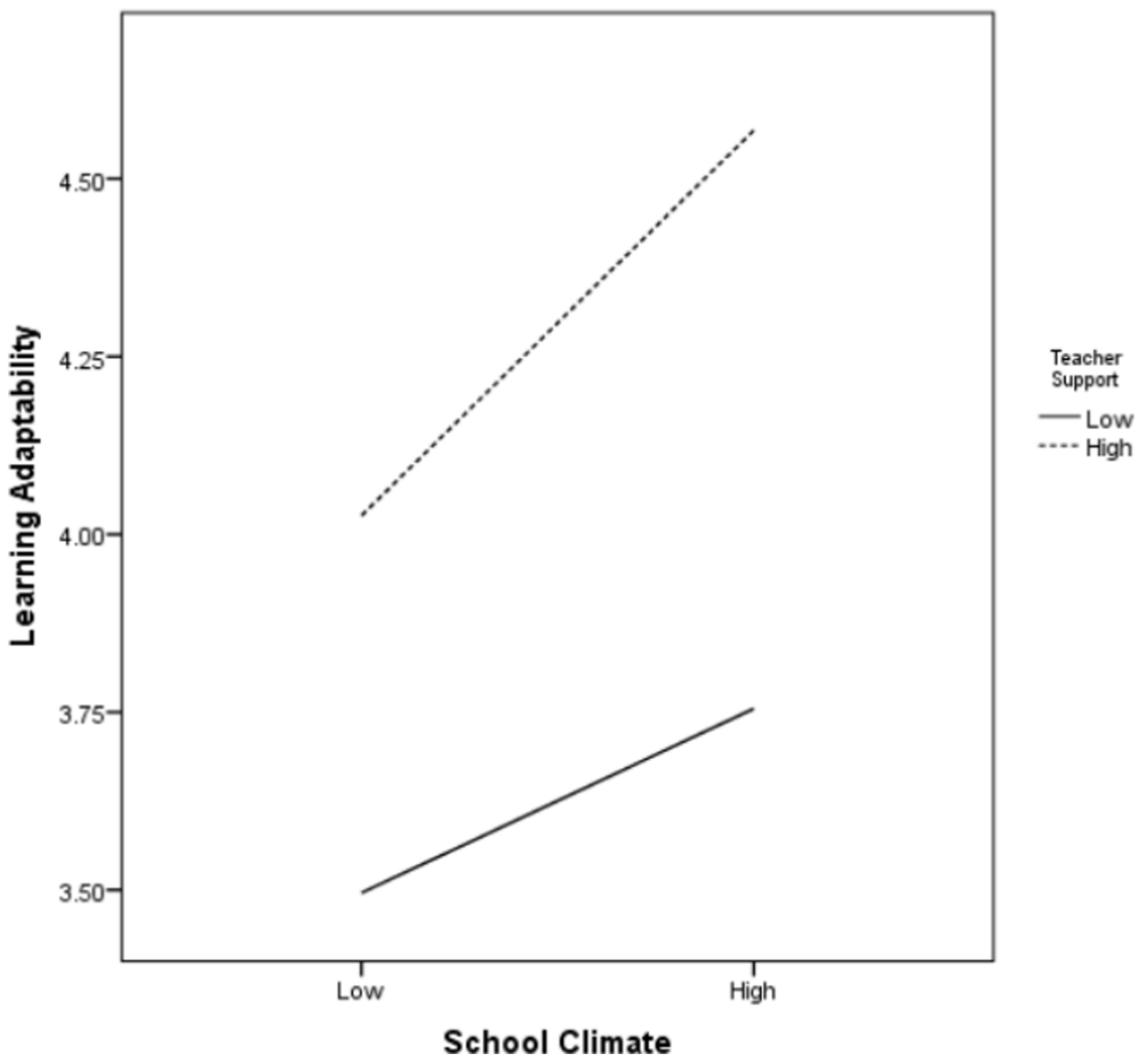
Moderating effects of teacher support on school climate and learning adaptability.

As some assumptions in the model involve mediating variables and moderating variables, this study used model 8 in the SPSS 24.0 macro program PROCESS version 3.5 to conduct conditional process analysis to verify whether teacher support mediates the relationship between school climate and left-behind children’s achievement motivation and the relationship between school climate and academic adjustment (Table 2). In order to ensure the reliability of analysis results and avoid problems in coefficient interpretation, the school climate and teacher support variables involved in the interaction item were set as mean-centered (Castro-González et al., 2019). The results showed that school climate had a positive impact on the learning adaptability of left-behind children (*a*1 = 0.338, *p* < 0.001), and the variable of learning adaptability also had a positive impact on the achievement motivation of left-behind children (*b* = 0.317, *p* < 0.001), substantiating H2 and H3. School climate had a direct influence on achievement motivation (*c*1 = 0.126, *p* < 0.001), which indicated that part of the influence of school climate on achievement motivation of left-behind children was not controlled by the learning adaptability of left-behind children, thus supporting H1. Therefore, in the influencing model of achievement motivation of rural left-behind children, school climate can directly affect achievement motivation on the one hand, and indirectly through the mediating variable learning adaptability on the other hand. That is, learning adaptability as a mediating variable holds. In addition, in the model with learning adaptability (M) as the dependent variable, the product interaction term between school climate and teacher support as the independent variable had a significant positive effect on the learning adaptability of left-behind children (*a*2 = 0.153, *p* < 0.001). It can be seen that teacher support moderates learning adaptability through school climate, substantiating H5. Moreover, teacher support as a moderating variable also moderates the relationship between school climate and achievement motivation (*c*2 = 0.061, *p* < 0.001), which supports H4. In order to more intuitively show the moderating effect of teacher support, this study defined the mean of the teacher support variable score, plus one standard deviation of the data as high teacher support data, and the sample mean minus one standard deviation of the data as low teacher support data. Thus, a simple slope effect diagram with different degrees of teacher support was obtained as shown in Figures 2, 3. Figure 2 shows that teacher support moderates the effect of school climate on left-behind children’s achievement motivation, with the effect of school climate on their achievement motivation growing along with the increase of teacher support. Figure 3 shows that school climate has a positive predictive effect on achievement motivation for students who perceive low-level teacher support; For students who perceive high-level teacher support, school climate still has a positive predictive effect on the development of achievement motivation, but the predictive effect is larger, which indicates that good teacher support can enhance the development of achievement motivation of left-behind children. As for the control variables, the gender and age of the control variables had a significant influence on the learning adaptability of the dependent variable in the learning adaptability model (*p* < 0.05). Gender had a significant positive effect on learning adaptability, while age had a significant negative effect on learning adaptability.

**Table 2:** Model coefficients for conditional process analysis.

Antecedents	*M* (Learning adaptability)			*Y* (Achievement motivation)		
	Coeff.	SE	*p*	Coeff.	SE	*p*
Constant	4.131	0.099	0.000	2.718	0.125	0.000
School climate	0.338^*a*1^	0.033	0.000	0.126^*c*1^	0.029	0.000
Teacher support	0.463	0.038	0.000	-	-	-
Learning adaptability	-	-	-	0.317*^b^*	0.023	0.000
School climate × Teacher support	0.153^*a*2^	0.030	0.000	0.061^*c*2^	0.025	0.016
Gender	0.054	0.027	0.043	0.005	0.023	0.832
Age	−0.015	0.007	0.040	−0.010	0.006	0.109
	*R*^2^ = 0.431, *F*(5, 1,411) = 214.028, *p* < 0.001			*R*^2^ = 0.350, *F*(6, 1,410) = 126.629, *p* < 0.001		

^*a*1^represents the effect value of school climate on learning adaptability; ^*a*2^represents the effect value of school climate and teacher support in the learning adaptability model; *^b^*represents the effect value of learning adaptability on achievement motivation; ^*c*1^represents the effect value of school climate on achievement motivation; ^*c*2^indicates the interaction between school climate and teacher support on achievement motivation.

**Figure 4:**
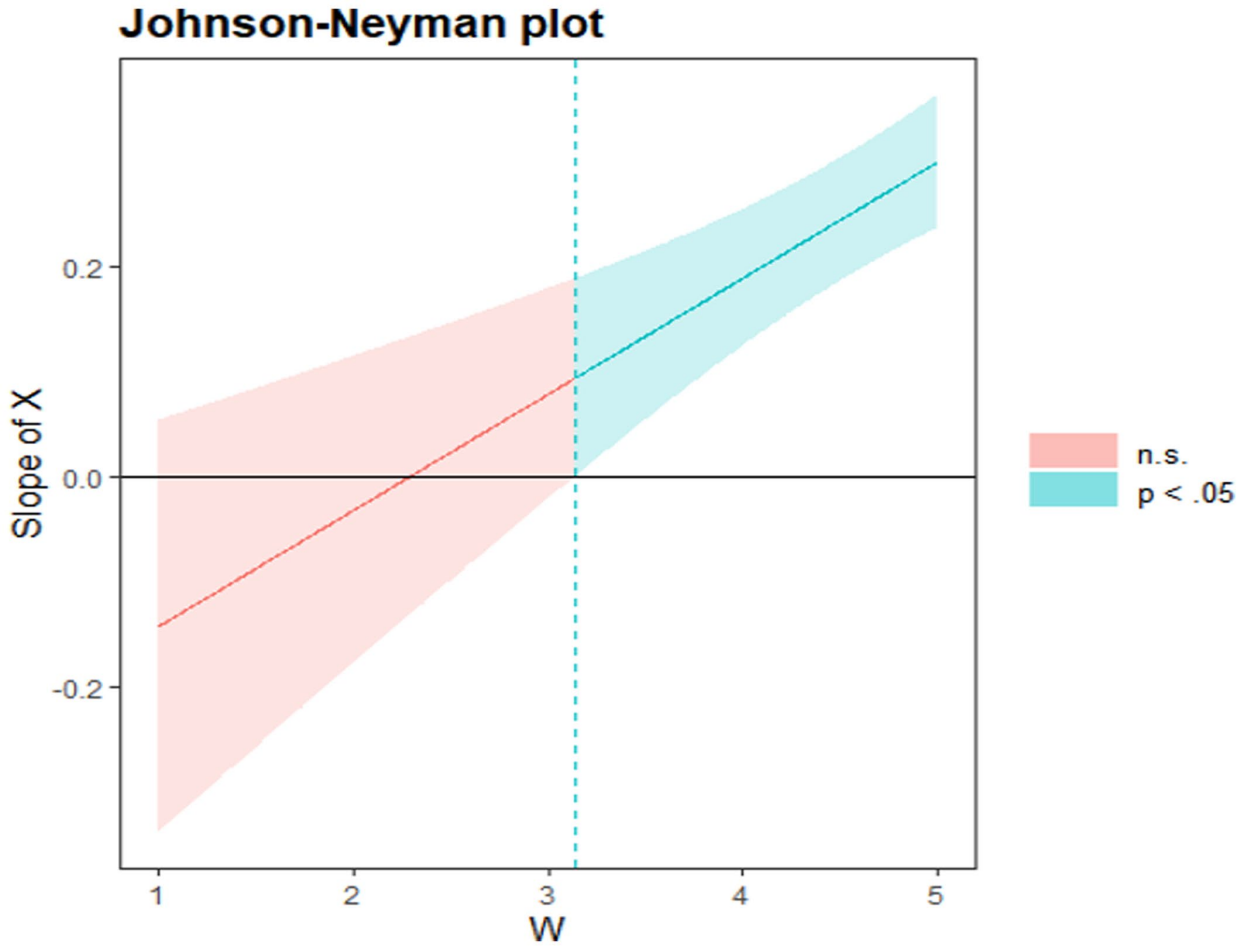
The moderating role of teacher support in the relationship between school climate and achievement motivation. *X* is the dependent variable school climate; *W* is the moderating variable teacher support.

**Figure 5:**
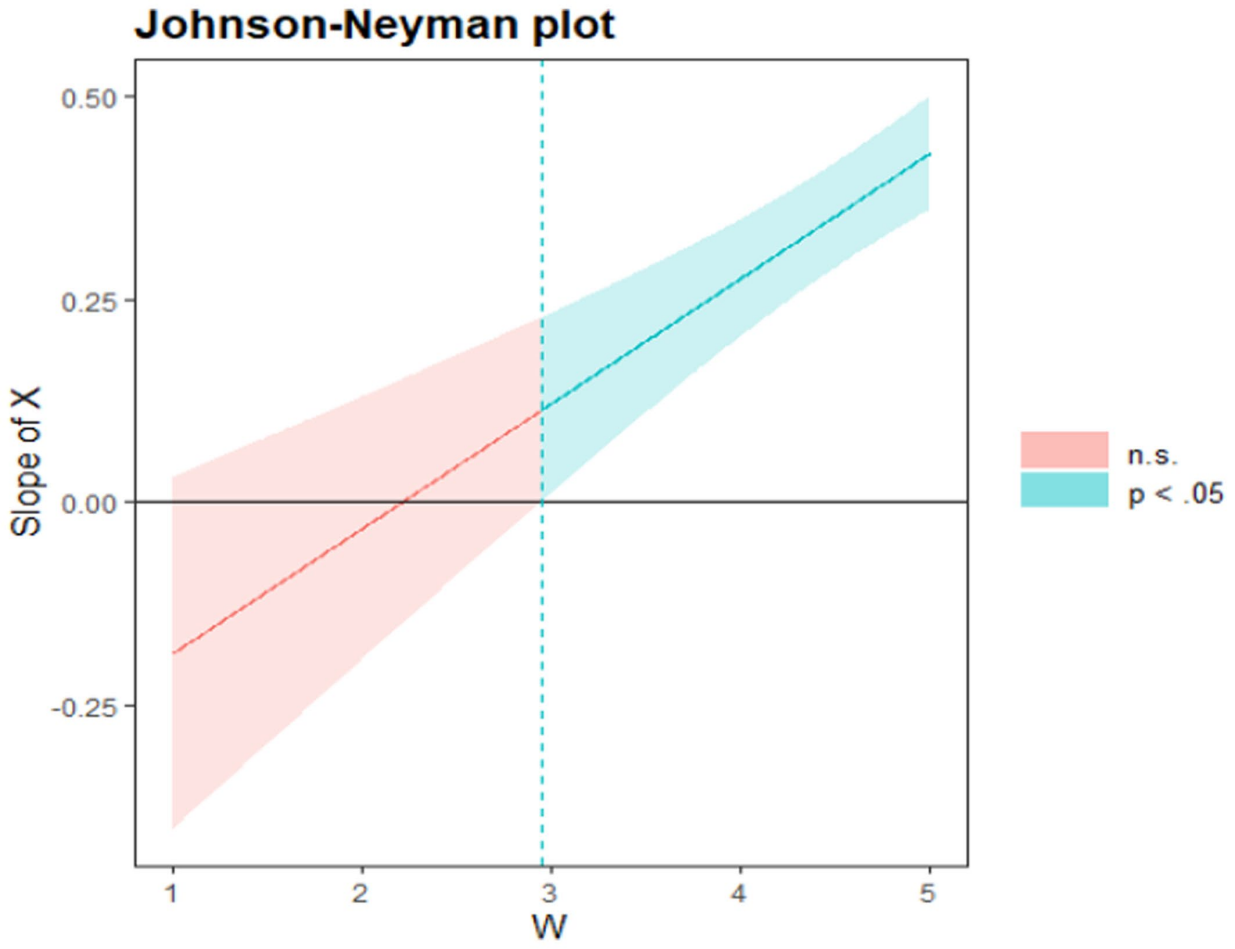
The moderating role of teacher support between school climate and learning adaptability. *X* is the dependent variable school climate; *W* is the moderating variable teacher support.

To verify the interaction between school climate and teacher support in the moderating variable model, this study adopted the point-selection method and the Johnson-Neyman method to test the simple slope (see Table 3; Figures 4, 5). Table 3 shows the direct and indirect effects of school climate on achievement motivation when the conditioning variables are set to different values. According to the statistical results, the amount of direct effect of school climate on achievement motivation varies with the value of the moderating variable. In addition, Table 3 lists the results of the direct and indirect impact analysis for the 10^th^, 25^th^, 50^th^, 75^th^, and 90^th^ percentile values, as well as the upper and lower limits of these effect confidence intervals. When the value of teacher support changed from the 10th percentile to the 90th percentile, the direct effect value changed from 0.072 to 0.160 and the indirect effect value changed from 0.064 to 0.134. Therefore, the higher the level of teacher support perceived by students, the greater the direct and indirect impact of school climate on achievement motivation. According to the analysis results of conditional indirect effects of school climate on achievement motivation in Table 3, when different values of teacher support are selected, the confidence interval did not contain zero, indicating that the indirect effects of school climate on achievement motivation were significantly positive, therefore, further supporting H2. However, this effect was not constant, and it was affected by the value of teacher support. This was consistent with the hypothesis that the higher the teacher support reflected by H5, the stronger the influence of school climate on achievement motivation, providing further supports to the mediating effect and the moderating effect. In addition, Table 3 supported the significant direct impact of school climate on achievement motivation. Therefore, the better the left-behind children’s perception of school climate was, the stronger their learning adaptability and achievement motivation would be. The ripple effect was even stronger for left-behind children who received more support from teachers. Figure 3 shows that when teacher support was outside the range of [0.090, 3.137], the simple slope line met the criterion of significance. At the same time, according to Figure 3, it can be found that the confidence interval of the simple slope did not contain zero and the slope was greater than zero, further supporting H4 that with the improvement of teacher support, the influence of school climate on learning adaptability was stronger and stronger. As shown in Figures 4, 5, the confidence intervals for the simple slopes of the two plots do not contain zero and have a slope greater than zero, further supporting H4 and H5 that the role of school climate in influencing achievement motivation increases as teacher support increases and the effect of school climate on learning adaptability becomes stronger.

**Table 3:** The direct and indirect effects of school climate on achievement motivation.

Moderator (teacher support)^a^	Effect	BootSE	CI
Conditional indirect effects of school climate on achievement motivation			
−0.882	0.064	0.025	[0.023, 0.120]
−0.438	0.086	0.019	[0.053, 0.127]
0.229	0.118	0.016	[0.088, 0.150]
0.562	0.134	0.018	[0.098, 0.170]
0.562	0.134	0.018	[0.098, 0.170]
Conditional direct effects of school climate on achievement motivation			
−0.882	0.072	0.038	[−0.002, 0.146]
−0.438	0.099	0.032	[0.037, 0.161]
0.229	0.140	0.029	[0.082, 0.197]
0.562	0.160	0.032	[0.098, 0.222]
0.562	0.160	0.032	[0.098, 0.222]
Index of moderated mediation			
	Index	BootSE	CI
	0.049	0.021	[0.006, 0.081]

a denotes values for the 10th, 25th, 50th, 75th, and 90th percentiles.

## 4. Discussion

As a preliminary exploration of achievement motivation of left-behind children aged 9–17 in mainland China, this study was the first to document the mediating effect of learning adaptability in the association between school climate and achievement motivation, as well as the moderating role of teacher support in this relation. Firstly, we found that the school climate of left-behind children in China had a significant positive impact on their achievement motivation. In the model, the relationship between the school climate and achievement motivation was affected by learning adaptability. Secondly, this study explored the dual moderating role of teacher support in the model. The results indicated that teacher support had a moderating effect between school climate and students’ achievement motivation and between school climate and students’ learning adjustment. We discussed each of our research questions in the following sections.

### 4.1. The mediating role of learning adaptability

We first tested the association between left-behind children’s perceived school climate and achievement motivation. Consistent with our expectations, left-behind children’s perceived school climate significantly predicted their achievement motivation. This finding was consistent with the findings of the available studies (Anderson, 1982; Alonso-Tapia and Ruiz-Díaz, 2022). We then examined the mediating effect of learning adaptability in this relation. Learning adaptability played a significant mediating role between the school climate and achievement motivation. According to the self-determination theory (Deci and Ryan, 2000b), human beings are positive organisms with innate tendencies for personal growth and the pursuit of happiness. This tendency is reflected in three basic psychological needs: autonomy, competence, and relatedness. The satisfaction of the three basic psychological needs of adolescents, autonomy, ability, and relationship, will promote the generation of their development behaviors. For left-behind children, a school climate characterized by high-quality learning resources, positive teacher-student relationships, and mutual aid to classmates could help students meet their basic needs (Tapia-Fonllem et al., 2020). Eventually, it will further encourage students to produce positive emotions to study and stimulate their achievement motivation, and prompt their desire for academic success.

Specifically, this study found that school climate had a positive predictive effect on left-behind children’s learning adaptability; In other words, when the school climate of left-behind children is better, their learning adaptability is higher. The results support the self-determination theory that the external learning environment can help children develop adaptive behaviors when it meets their internal psychological needs. Studies (Hunt, 1975; Jones et al., 2020) have shown that when there is a positive student-teacher relationship and a teaching climate that stimulates students’ interest, students are more likely to find a sense of belonging to the school environment and can participate more actively in learning and develop emotional and social skills (Leurent et al., 2021). For left-behind children, the school environment and children’s social relations in school become the main sources of their needs for a sense of belonging and a sense of achievement because their parents are away all year round. When children perceive that the school climate matches their needs, it will affect their understanding of themselves and the formation of their attitudes, thus improving their learning adaptability (Lara and Saracostti, 2019).

In the mediation model of learning adaptability, we discovered that learning adaptability was positively correlated with achievement motivation of left-behind children, and that learning adaptability had obvious predictive power and significant influence on students’ achievement motivation. Learning adaptability has been reported to play an important role in educational settings, predicting higher classroom participation, higher academic achievement, and higher life satisfaction (Collie et al., 2017; Burns et al., 2018). The results of this study further proved that learning adaptability is a key factor influencing the achievement motivation of left-behind children. On the one hand, the emergence of this research conclusion is related to the successful experience of left-behind children. Children with strong learning adaptability are more able to overcome difficulties in the learning process, make various adaptive activities adjust the learning process, and then obtain successful experiences. The acquisition of successful experiences can help children build confidence and increase their expectations of success (Grund et al., 2022). On the other hand, this conclusion may also be related to the left-behind environment of left-behind children. Family poverty contributes to the resilience of left-behind children’s life and learning attitudes. They hope to get out of their current predicament and change their future by improving their education levels. As a result, they tend to show a strong motivation to achieve in learning.

### 4.2. The moderating role of teacher support

This study found that, after controlling for age and gender, teacher support had a significant moderating effect on the relationship between school climate and achievement motivation and on the relationship between school climate and learning adjustment. Meanwhile, when the intensity of teacher support increases, the effect of school climate on the achievement motivation of left-behind children increases, as does the effect of school climate on children’s adjustment to learning. Specifically, school climate had a stronger effect on achievement motivation and learning adjustment in left-behind children who experienced high levels of teacher support than left-behind children who experienced low levels of teacher support.

An in-depth analysis of the research results shows that the moderating effect of teacher support in the mediation model is consistent with the risk and resilience framework, and the protective factors may weaken the adverse effects of risk factors (Suárez-Orozco et al., 2018). In other words, when faced with a low-level school climate, higher teacher support can help left-behind children offset the impact of a bad school climate on their achievement motivation. When facing a higher-level of school climate, higher teacher support can improve the development of achievement motivation of left-behind children based on meeting their needs for autonomy. The reason lies in that teachers, as important guides in the growth of left-behind children are important subjects of responsibility in school education. Due to the alienation phenomenon of the parent–child relationship of left-behind children caused by their parents working outside, it is easy to cause the loss of family function. As the main contact object of left-behind children, school teachers can replace their parents’ responsibilities to a certain extent. Teachers’ support and encouragement to children in learning can help children form good learning habits and maintain good learning attitudes in the learning process, thus promoting the development of achievement motivation of left-behind children (Lee and Simpkins, 2021; Pap et al., 2021).

In the hypothesized model, the moderating point of teacher support played a role in the first half of the mediation effect, that is, the relationship between school climate and left-behind children’s learning adaptability depends on the level of teacher support. According to the protective-protective model (Christiansen and Evans, 2005), the predictive power of one protector factor on the outcome may vary with the level of the other. In the current study, the protective factors are school climate and teacher support, and the outcome variable is learning adaptability, that is, the predictive effect of school climate on learning adaptability will change with the level of teacher support. For left-behind children, the perceived high level of teacher support can buffer the negative impact of adverse family factors on their non-cognitive ability (Lei and Li, 2020), thus having a positive impact on their learning life, mental health, and personal development, enabling them to maintain a good learning attitude and promote the development of their learning ability. In addition, control variables were set up to eliminate the interference of gender and age of left-behind children in the model. The results show that, based on controlling gender and age, there is a significant moderating effect between school climate and learning adaptability of left-behind children. As an important responsibility subject in the social support system for left-behind children, teachers’ supporting role can adjust the learning adaptability of left-behind children to promote the development of their achievement motivation. Left-behind children’s perceived teacher support will have different effects on their learning adaptability and achievement motivation. When left-behind children perceive a high level of teacher support, they are more willing to seek help from teachers when they encounter difficulties and obstacles in life or learning, to solve problems, and promote personal achievements and development.

## 5. Contribution and implications

This study has the following contributions. First of all, this study concentrates the current research attention on the group of left-behind children and enriches the research category of achievement motivation. Achievement motivation is not only related to students in higher education, but it is also closely related to left-behind children who are primary recipients of benefits associated with the cultivation of achievement motivation. Acknowledging the importance of achievement motivation research on left-behind children is an important prerequisite for the future study of the influence mechanism of achievement motivation. Secondly, this study explores achievement motivation, school climate, learning adaptability, teacher support status quo, and their relationship among left-behind children, and makes an in-depth analysis of the mechanism of learning adaptability and teacher support between left-behind children’s perceived school climate and achievement motivation. This can provide empirical support for future research on the underlying mediating and moderating mechanisms between achievement motivation and the school climate, of left-behind children.

This study carries important implications for the cultivation of achievement motivation of left-behind children. Firstly, this finding suggests that a positive school climate can improve children’s learning adaptability and thus promote achievement motivation of left-behind children. Therefore, schools should pay attention to the construction of a strong learning atmosphere, the establishment of a harmonious relationship between teachers and students, and the effective supply of rich learning resources to help left-behind children establish a healthy learning attitude and master more learning abilities. Secondly, this finding indicates that more supportive help provided by teachers for left-behind children will help children better benefit from the school environment. Therefore, teachers should provide more supportive feedback and emotional attention to left-behind children, and help them correct their learning attitudes, improve their learning abilities, and form good learning habits. Thirdly, as the subject of school education with professional knowledge such as psychology and career planning, school consultants should give full play to their professional advantages. School counselors can use the skills and tools of psychological counseling to provide left-behind children with individual counseling services in learning, psychology and other aspects, discover the problems behind children’s external behavior and guide students to change their behavior habits. In addition, school counselors can design career planning activities, provide professional services for children, and help left-behind children establish a better self-awareness, find out what jobs they are more suitable for and what jobs they will do in the future, so as to stimulate the awareness of self-reliance among left-behind children. Fourthly, the learning adaptability of left-behind children can promote the development of their achievement motivation. Thus, left-behind children should not lose their learning enthusiasm and thus reduce their achievement motivation in the absence of their parents. In addition, left-behind children should have a clear understanding of themselves, establish a correct view of learning, discover the fun in learning, and improve their learning ability and enthusiasm.

## 6. Limitations and future research

However, the current study has several major limitations. Firstly, this study adopts a cross-sectional study, which makes it difficult to derive the causal relationship between variables. In future studies, longitudinal studies can be used to provide more powerful arguments to demonstrate the influence of school climate on the achievement motivation of left-behind children. Secondly, the measurement of school climate, teacher support, learning adaptability, and achievement motivation were all self-reported data, limited by recall bias and social expectations. Future research should collect various data types, such as surveys and interviews of teachers and parents of left-behind children, and collect relevant information about left-behind children from side to increase the reliability of results. The observation method can provide more objective and real data to reveal the school environment and behavior of children, while the interview method can provide researchers with in-depth information about the school atmosphere and achievement motivation of left-behind children. Finally, this study only investigated the left-behind children in one province in western China, whose behavioral characteristics and school status may be different from those in other parts of China and other countries, thus limiting the generalizability of the research results. Therefore, future research can consider adding different samples of left-behind children. At the same time, this study investigates the learning atmosphere as an overall dimension, and it can also consider investigating the influence of different school atmospheres on the achievement motivation of left-behind children.

## 7. Conclusion

To sum up, this study investigated the moderating mediation model of the relationship between left-behind children’s perception of school climate and their achievement motivation. Our findings identified the underlying mechanisms behind this relationship (i.e., mediating learning adaptability and moderating teacher support), which contributes to our understanding of the key processes by which a positive school climate prompts achievement motivation in left-behind children. Learning adaptability can serve as a potential intermediary to making good school climate work, which in turn promotes achievement motivation. A focus on children’s learning adaptability will bring additional nuances to children’s perception of the link between school climate and achievement motivation. In addition, our findings indicated that teacher support moderates the direct relationship between school climate and learning adaptability, and left-behind children with high perceived teacher support had higher learning adaptability than left-behind children with low perceived teacher support. Finally, under a certain school climate, children with high perceived teacher support had a higher positive impact on achievement motivation through improving their learning adaptability than left-behind children with low perceived teacher support. Therefore, the findings of this study have considerable implications for the development and improvement of achievement motivation of left-behind children.

## Data availability statement

The raw data supporting the conclusions of this article will be made available by the authors, without undue reservation.

## Ethics statement

The studies involving human participants were reviewed and approved by The Ethics Committee of Qufu University. Written informed consent to participate in this study was provided by the participants’ legal guardian/next of kin.

## Author contributions

KZ designed the study, helped with data collection, and analyzed the data. NC and GL designed the study, analysed the data, and wrote the manuscript. ZL and XW helped with data analysis and edited the manuscript. All authors contributed to the article and approved the submitted version.

## Funding

This research was supported by the General Project of National Social Science Foundation of China (grant number 20BSH052) and Taishan Scholar Project of Shandong Province.

## Conflict of interest

The authors declare that the research was conducted in the absence of any commercial or financial relationships that could be construed as a potential conflict of interest.

## Publisher’s note

All claims expressed in this article are solely those of the authors and do not necessarily represent those of their affiliated organizations, or those of the publisher, the editors and the reviewers. Any product that may be evaluated in this article, or claim that may be made by its manufacturer, is not guaranteed or endorsed by the publisher.
